# Reduced Synaptic Vesicle Recycling during Hypoxia in Cultured Cortical Neurons

**DOI:** 10.3389/fncel.2017.00032

**Published:** 2017-02-16

**Authors:** Sergei Fedorovich, Jeannette Hofmeijer, Michel J. A. M. van Putten, Joost le Feber

**Affiliations:** ^1^Laboratory of Biophysics and Cellular Engineering, Institute of Biophysics and Cell Engineering, National Academy of Sciences of BelarusMinsk, Belarus; ^2^Clinical Neurophysiology, University of TwenteEnschede, Netherlands; ^3^Department of Neurology, Rijnstate HospitalArnhem, Netherlands; ^4^Department of Neurology and Clinical Neurophysiology, Medisch Spectrum TwenteEnschede, Netherlands

**Keywords:** synapse, endocytosis, exocytosis, hypoxia, brain, styryl dye, cultured neurons

## Abstract

Improvement of neuronal recovery in the ischemic penumbra, an area around the core of a brain infarct with some remaining perfusion, has a large potential for the development of therapy against acute ischemic stroke. However, mechanisms that lead to either recovery or secondary damage in the penumbra largely remain unclear. Recent studies in cultured networks of cortical neurons showed that failure of synaptic transmission (referred to as synaptic failure) is a critical factor in the penumbral area, but the mechanisms that lead to synaptic failure are still under investigation. Here we used a Styryl dye, FM1-43, to quantify endocytosis and exocytosis in cultures of rat cortical neurons under normoxic and hypoxic conditions. Hypoxia in cultured cortical networks rapidly depressed endocytosis and, to a lesser extent, exocytosis. These findings support electrophysiological findings that synaptic failure occurs quickly after the induction of hypoxia, and confirms that the failing processes are at least in part presynaptic.

## Introduction

Stroke is the third leading cause of death and the second largest cause of chronic adult disability in the Western World. About 85% concerns a brain infarct, resulting from blockage of arterial blood flow to the brain. In the core of a brain infarct, blood supply is usually not enough to maintain ion gradients across the neuronal plasma membrane, and loss of neuronal function is followed by neuronal death within minutes. Otherwise, in peripheral areas of an infarct, the so called penumbra, damage is not (yet) irreversible due to blood supply from surrounding arteries. In the penumbra, neurons are functionally silent, but structurally intact and viable. These may eventually recover, or proceed to cell death, but the mechanisms behind these diverging scenarios are not clearly understood. Therapies to prevent collateral damage of penumbral brain tissue have a large potential to improve neurological outcome of patients with brain infarcts, but are lacking (George and Steinberg, 2015).

As an early consequence of cerebral ischemia synaptic activity is lost, and failure of synaptic transmission (referred to as synaptic failure) has been proposed to account for electric silence in the penumbra (Hofmeijer and van Putten, 2012; Hofmeijer et al., 2014; le Feber et al., 2016). The changes of synaptic functioning are generally assumed to be reversible (Gao et al., 1999; Bolay et al., 2002; Hofmeijer et al., 2014; le Feber et al., 2016). It is even hypothesized that suppression of functional synaptic activity may reflect a compensatory mechanism that restores the balance between oxygen supply and consumption in favor of maintaining resting potentials and thus preserves the neurons' structural integrity (Hochachka et al., 1996). On the other hand, persisting synaptic failure has been associated with progression toward irreversible neuronal damage, even in the absence of membrane depolarization. Most evidence supports the notion that ischemic synaptic failure occurs primarily at the presynaptic terminal and that it involves impaired transmitter release (for a review see Hofmeijer and van Putten, 2012). However, details on the molecular mechanisms are unclear.

Here, we investigated synaptic vesicle recycling, a presynaptic process that seems plausible to play an important role in hypoxia induced synaptic failure. Under normal conditions, synaptic transmission is initiated when an action potential arrives at the presynaptic nerve terminal, inducing the opening of voltage gated Ca^2+^ channels. The resulting temporary increase of intracellular Ca^2+^ triggers synapsin phosphorylation, which releases vesicles from the reserve pool to the ready releasable pool to enable exocytosis. After exocytosis, synaptic vesicles are recycled by endocytosis and refilled with neurotransmitter (Dittman and Ryan, 2009). At least several steps of the synaptic vesicle cycle are strongly ATP-dependent, including neurotransmitter loading, disassembling of SNARE complexes, priming, and endocytosis (Südhof, 2004, 2013). It is unknown, what exact parts of the synaptic vesicle cycle are affected by hypoxia. This is difficult to investigate using electrophysiological tools alone. Imaging of styryl dyes (for instance, FM 1–43) allows for separate visualization of various steps of the synaptic vesicle cycle (Cochilla et al., 1999). Here we investigated the effect of hypoxia on endocytosis and exocytosis using the fluorescent dye FM1-43.

## Methods

### Cell cultures

We obtained cortical neurons from new-born Wistar rats on the day of birth. All procedures involving animals were conducted according to Dutch and European laws and guidelines, and approved by the Dutch Animal Use Committee (DEC). After trypsin treatment, cells were dissociated by trituration. About 100,000 dissociated neurons were plated on a coverslip or on a multi electrode array (MEA; 60 electrodes with a 30 μm diameter and 200 μm pitch; Multi Channel Systems, Reutlingen, Germany), both pre-coated with poly ethylene imine (PEI). To enable comparison to earlier electrophysiological measurements, we used the same high cell density as previous work (Hofmeijer et al., 2014; le Feber et al., 2016). Cells were plated at an initial density of approximately 5,000 cells per mm^2^, with aging, cell densities gradually decreased to ~2,500 cells/mm^2^.

Neurons were obtained in four different preparations and cultured on coverslips (*n* = 26) stored in 24-well plates or in a circular chamber glued on top of the MEA (*n* = 2). The culture chambers were filled with R12 medium (Romijn et al., 1984) and all cultures were stored in an incubator, under standard conditions of 36°C, 100% humidity, and 5% CO_2_ in air. We kept cells in culture for at least 3 weeks, allowing for the development of mature networks. Medium was refreshed twice a week (300 μL of old medium was replaced by 400 μL fresh medium).

For the induction of hypoxia, we placed the cultures under a Plexiglas hood, referred to as hypoxic chamber, where under a continuous flow of a computer regulated mixture of air and nitrogen was kept. Five % CO_2_ was added to the gas mixture and humidity was maintained. Cultures on coverslips were put in the hypoxic chamber in 24-well plates, the MEA culture chambers were sealed with watertight but O_2_ and CO_2_ permeable foil (MCS; ALA scientific). For experiments, cultures were transferred to a confocal microscope (Zeiss LSM 510). Experiments began after an accommodation period of at least 20 min. Immediately before the start of an experiment covers were removed from the culture chambers to optimize visual access and to facilitate quick medium changes. This allowed oxygen to re-enter the medium, which occurred at a relatively slow rate (see below). From that point, maintenance of sterility was not necessary anymore, because experiments typically lasted less than 10 min.

#### Hypoxia

Prior to the measurements, cultures were exposed to hypoxia during 6 h. This was achieved in the hypoxic chamber by replacing 90% of air by nitrogen, which yielded a lowering of partial oxygen pressure (pO_2_) from pO_2_ ≈160 mmHg to pO_2_ ≈20 mmHg. Partial oxygen pressures were measured using an optical oxygen sensor (PHOSPOR, Ocean Optics). Earlier electrophysiological measurements clearly showed synaptic failure during 6 h of hypoxia at this depth (le Feber et al., 2016). All solutions used for imaging were also kept in the hypoxic chamber to obtain equal pO_2_, prior to administration. Besides controlled hypoxia, the hypoxic chamber allowed keeping the neurons under standard conditions (36°C, 100% humidity, and 5% CO_2_). Control coverslips (without hypoxia) were incubated in a CO_2_ incubator under standard conditions.

**Figure 1:**
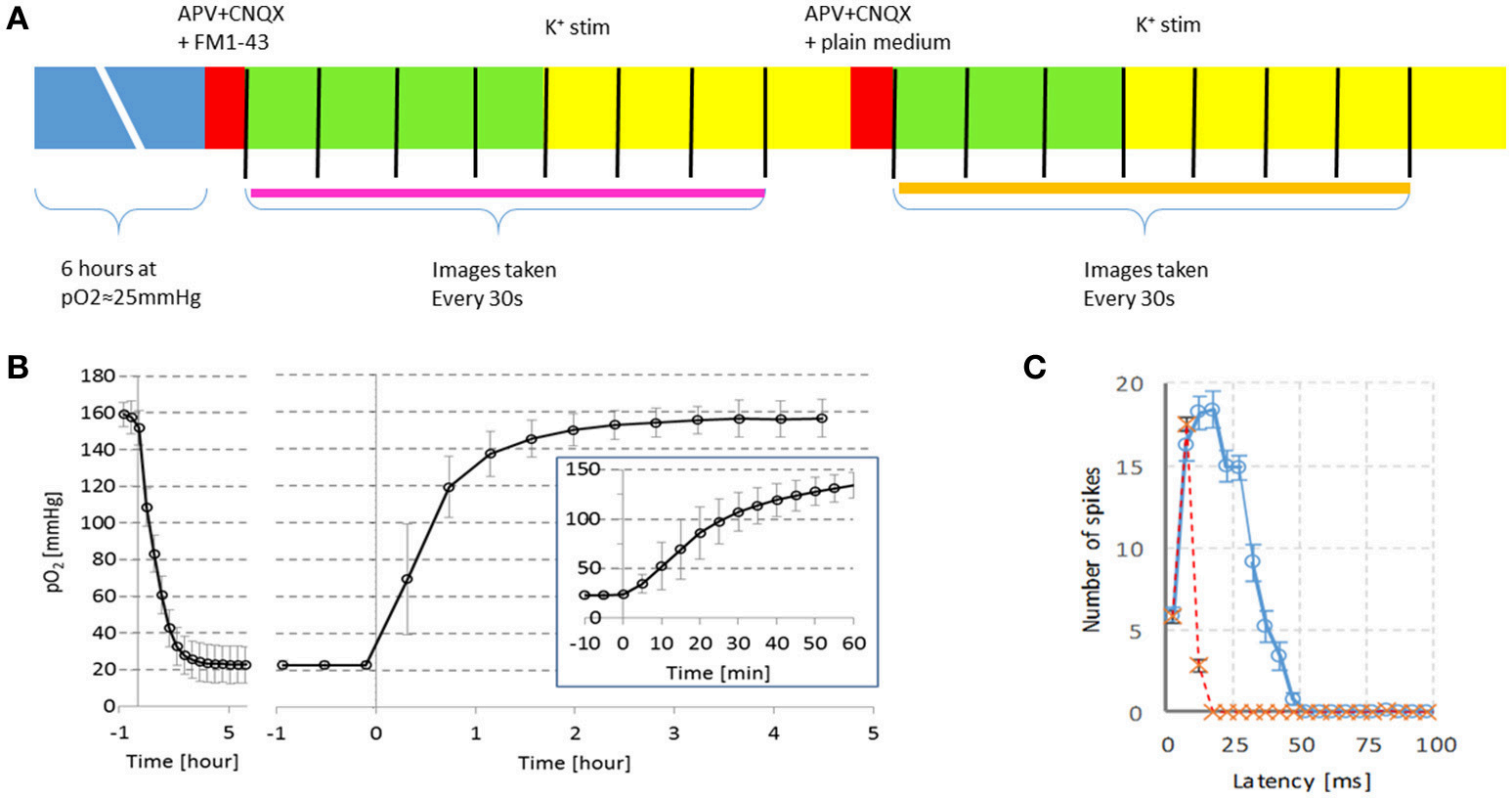
**Timeline of the experiments and verification of experimental conditions. (A)** Cultures were exposed to 6 h of hypoxia at pO_2_ ≈20 mmHg. Then, excitatory synaptic transmission was blocked by APV and CNQX and an FM dye was added to the medium. The cultures were installed under the microscope and imaging started (every 30 sec, indicated by vertical black lines). Cultures were stimulated in the 2nd min of imaging by potassium (*n* = 26) or electrically (*n* = 2). Electrical stimulation was repeated in the 4th min of imaging. Then the FM dye was washed out, and cultures were stimulated again in the 2nd min after medium change. Endocytosis measurement is indicated by the thick pink line, exocytosis measurement is indicated by the orange line. **(B)** Partial oxygen pressure (pO_2_) in culture medium with a cortical culture (mean ± *SD* of *n* = 3) following a stepwise change in gas mixture settings at *t* = 0. Left panel: The gas mixture fed to the hypoxic chamber was changed from normoxia (pO_2_ ≈160 mmHg) to hypoxia (pO_2_ ≈20 mmHg) at *t* = 0. Right panel: At *t* = 0 the gas mixture returned to normoxia. Before and after the oxygen measurements, electrical activity was recorded from the neurons, confirming that the cells were alive. Inset: pO_2_ during the 1st h at higher temporal resolution. **(C)** Example of responses to electrical stimulation before (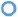, solid blue line), or after (x, dashed red line) blockade of glutamatergic synaptic transmission. All recorded action potentials are counted in 5ms bins after each stimulus pulse, mean ± SEM of 10 stimulus pulses are shown. In both cultures the late phase of the stimulus response (latency >15 ms) was completely blocked at the concentrations used.

After this incubation period (exposure to hypoxia) the medium of coverslips was changed to medium containing ionotropic receptor inhibitors 50 μM DL-2 amino-5-phosphonovaleric acid (APV; a selective blocker of the NMDA glutamate receptor; Sigma-Aldrich) and 10 μM 6-cyano-7-nitroquinoxaline-2,3-dione (CNQX; a selective AMPA receptor blocker; Sigma-Aldrich), which had been exposed to the same hypoxic conditions. Figure 1A illustrates the experimental protocol. In the two cultures plated on MEAs, we recorded spontaneous activity and responses to electrical stimulation as described in le Feber et al. (2016). These cultures were used to verify the efficacy of excitatory blockade at this concentration by evaluation of their responses to electrical stimulation. Two types of imaging solutions were used in different experiments: R12 cell culture medium and the colorless modified Tyrode solutions (136 mM NaCl, 2.5 mM KCl, 10 mM HEPES, 10 mM glucose, 1.3 mM MgCl_2_, pH 7.4), to verify that the color of R12 did not interfere with detection of the FM dye.

### Imaging

Endocytosis and exocytosis were imaged using a fluorescent dye FM1-43 (Sigma-Aldrich) according to Stevens and Williams (Stevens and Williams, 2000) and a confocal microscope (LSM 510, Carl Zeiss) with objective 20x 0.5 n.a. for experiments with electrical stimulation, or with 40x 0.6 n.a. objective (LD Achroplan, Carl Zeiss) for experiments with potassium stimulation. It was shown that this magnification is sufficient for synaptic vesicles recycling imaging (Klingauf et al., 1998; Stevens and Williams, 2000; Rangaraju et al., 2014). The glass ring on the MEAs impeded larger magnification than 20x because larger objectives could not get close enough to the sample.

Due to the acute nature of experiments we could not photograph many areas sequentially. Therefore, we used a large scanning area and consequently, the scanning resolution was relatively low (scanning area and scanning resolution are reciprocally related on our confocal, if the total time for imaging is fixed). For quantification of the effect of hypoxia on endo- and exocytosis, reproducibility of changes is crucial. Lower resolution introduces more noise in the images and may thus reduce reproducibility. However, the chosen resolution was sufficient to observe significant differences.

Images were acquired every 30 s. The dye was excited by an argon laser (488 nm) while collecting the emission through a 505 nm long-pass filter. Two strategies for dye loading and releasing were used in different experiments: electrical and potassium stimulation (see below). In both cases, 10 μM of FM1-43 was added to the incubation medium.

The R12 culture medium contains a pH indicator, and has a slight purple color at healthy pH. To verify that this color did not interfere with imaging, we compared the amplitude of fluorescence changes in R12 with that in modified Tyrode solution, and found no differences (data not shown).

All other images were collected in R12 cell culture medium.

### Neuronal stimulation to induce endocytosis and exocytosis

**Figure 2:**
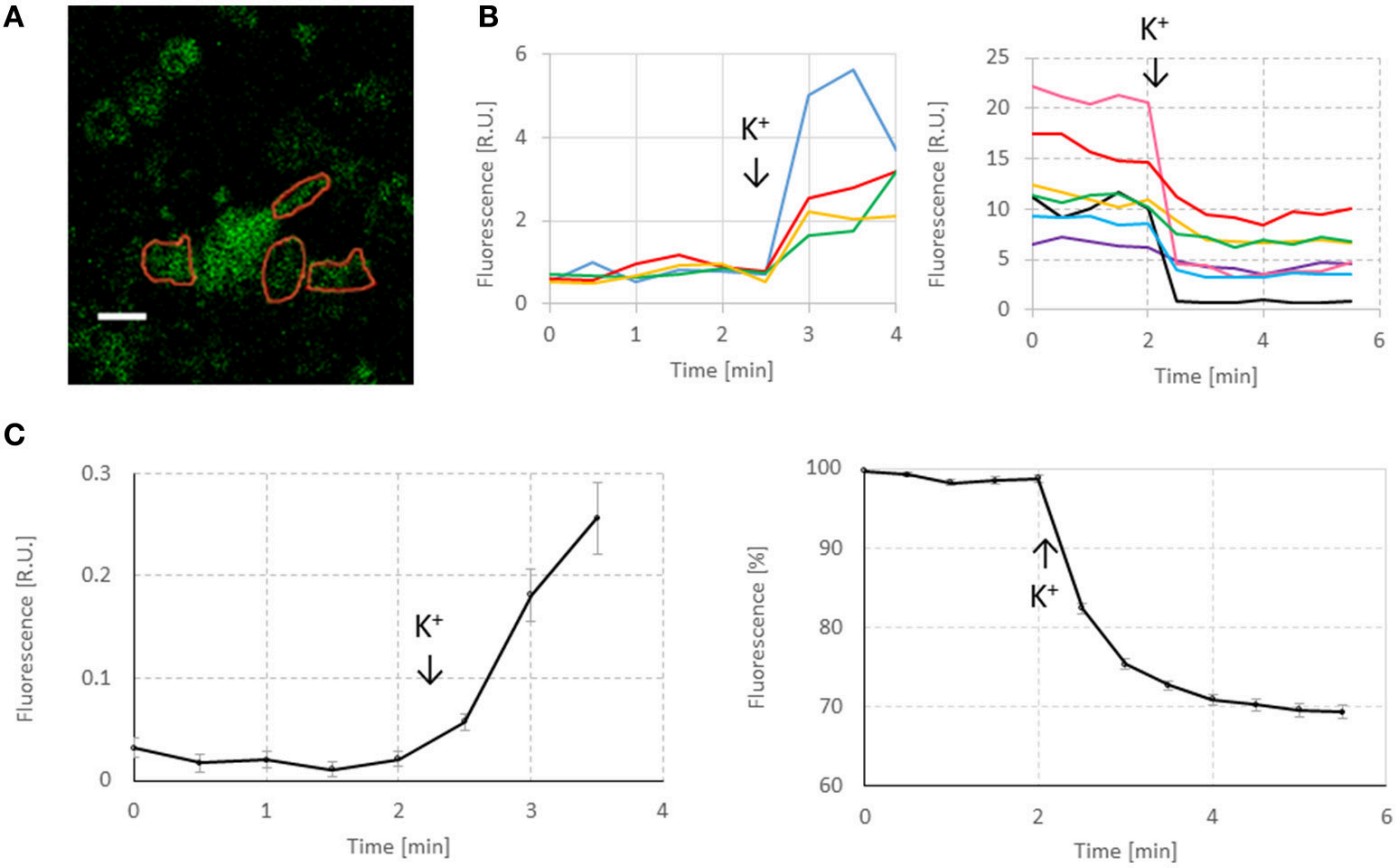
**Representative responses of FM1-43 in neurons. (A)** Image taken before exocytosis. Red lines indicate selected ROIs. White scale bar: 10 μm. The imaging resolution is somewhat low, as a consequence of maximizing the scanning area. However, it was sufficient to quantify the effect of hypoxia on endo- and exocytosis and to observe significant differences between normoxia and hypoxia. **(B)** Representative responses to first potassium stimulation and to second potassium stimulation after washing. First increase in fluorescence was used to quantify endocytosis. The second response (decreased fluorescence) was used to quantify exocytosis. Addition of 60 mM KCl was indicated by arrows and “K^+^.” **(C)** Average endocytosis and exocytosis curves under control conditions (*n* = 258 ROIs in 11 cultures). For endocytosis curves, minimum fluorescence before stimulation was subtracted from all points to facilitate averaging. Exocytosis curves were normalized to their fluorescence at 2 min before stimulation.

All cultures on coverslips were stimulated by adding 60 mM KCl in the 2nd min of imaging. If modified Tyrode solution was used, 2 mM of CaCl_2_ was added in parallel. Before stimulation, there was no fluorescence. Increased fluorescence at 1.5 min after potassium addition was considered to reflect endocytosis (Gaffield and Betz, 2006). This relatively long period was necessary because endocytosis was relatively slow in our experiments. We could not use short pulses of high potassium concentration due to the absence of a perfusion system. Figure 2B shows a typical response to first K^+^ stimulation to load the dye. Then, medium was changed to wash out the dye and again potassium was added in the 2nd min of imaging. Decreasing fluorescence during minutes 2–4 after the medium change was considered to reflect exocytosis (see Figure 2C). Background fluorescence in the 2nd min was set to 100%, and all fluorescence intensities were normalized to this value to facilitate averaging across cultures. Due to the absence of fluorescence before stimulation, such normalization was not possible for the quantification of endocytosis. The 2 cultures on MEAs were electrically stimulated to image endo- and exocytosis, but these results were not used in the current study.

Figure 1A summarizes the experimental protocol.

### Analysis

Images were analyzed using ImageJ. Regions of interest (ROIs) were placed manually around individual puncta or small groups of nearby situated puncta that seemed to respond to stimulation, see Figure 2A. The modus of imaging (endocytosis or exocytosis) was known to the researcher that selected the ROIs, but not the normoxic or hypoxic conditions. Selection of ROIs consisted of three steps.

1. Selection of ROI. In case of endocytosis boutons (group of puncta) with increase of fluorescence were selected, in case of exocytosis boutons with decrease of fluorescence were selected. Very few boutons responded the opposite way (this was only possible in case of exocytosis due to the very low baseline fluorescence in experiments with endocytosis). Locations with unchanging fluorescence were classified as non-specific.
2. Selection of curves. Curves with unstable initial fluorescence were discarded, as well as curves with a “response” onset that differed from the time of stimulation.
3. Selection of amplitudes. Responses that differed more than 2 SD from the averaged response were discarded. Generally, these were boutons with 100% exocytosis, which most likely were neurons that lysed in response to high potassium.

In all ROIs we calculated the maximum change in fluorescence. Increasing fluorescence during endocytosis is expressed in arbitrary units (R.U.). Minimum fluorescence before stimulation (background fluorescence) fluctuated slightly and was subtracted before averaging across ROIs.

Decreasing florescence during exocytosis is expressed as a fraction of the fluorescence after dye loading to compensate for possible, hypoxia induced, baseline fluorescence differences immediately after loading of the dye.

Data are presented as mean ± S.E.M unless stated otherwise. Where indicated, statistical significance was evaluated by two-tailed Student's *t*-test. Normality of data was confirmed by the Shapiro-Wilkinson test (OriginPro 8).

## Results

Two cultures plated on MEAs were spontaneously active, and showed synchronous patterns, commonly referred to as bursts. In both cultures, electrical stimulation effectively induced a network response before blockade of glutamatergic synaptic transmission, whereas application of APV and CNQX impeded the second, synaptically mediated phase of the response (Figure 1C). Electrical stimulation confirmed the glutamatergic blockade at the used concentrations of APV and CNQX, but could no longer excite a sufficiently large number of neurons, necessary to quantify endocytosis and exocytosis at a network scale. Therefore, all further experiments applied potassium stimulation.

### Effect of hypoxia on the synaptic vesicle cycle

We performed experiments with potassium stimulation in R12 cell culture medium under normoxic or hypoxic conditions. After exposure to hypoxia, before imaging, cultures were transferred to the confocal microscope and hypoxic conditions were no longer maintained. We found that when hypoxic solutions were transferred to open air, hypoxic depth slowly decreased, but the oxygen level remained far below normoxia, at least during the next 12 min (see Figure 1B, inset).

To quantify endocytosis and exocytosis, we selected ROIs around synaptic boutons that seemed to respond to stimulation. Figure 2 shows some examples of fluorescence changes following potassium stimulation. Non-specific decrease of fluorescence in the curves before potassium stimulation varied between ROIs (see Figure 2B), but always remained in the range 0–10% (<2 R.U. in case of excocytosis), suggesting that photo bleaching did not substantially affect results.

Infrequently, ROIs showed increasing fluorescence during exocytosis measurements under normoxic (*n* = 41; 5.7%) or hypoxic conditions (*n* = 38; 6.2%). These ROIs were not selected for further analysis. Notably, most of these ROIs did not pass the second inclusion criterion either (nonstable baseline and/or fluorescence increased before stimulation). The selected ROIs covered about 50–80% of the imaged areas. This percentage depended on the cell density, which slightly differed between cultures, and even between different locations within cultures. About 20% of all initially chosen ROIs were excluded from analysis because they showed no response to stimulation or because of unstable baseline fluorescence, see Table 1.

**Table 1:** **Included ROIs**.

**Endocytosis**				**Exocytosis**			
**Control (11 coverslips from 4 preparations)**		**Hypoxia (8 coverslips from 4 preparations)**		**Control (14 coverslips from 4 preparations)**		**Hypoxia (12 coverslips from 4 preparations)**	
**Total: 352**	**Included: 258**	**Total: 110**	**Included: 101**	**Total: 679**	**Included: 526**	**Total: 580**	**Included: 452**
73%		92%		77%		78%	

*ROIs were selected as explained in Methods. Several ROIs were discarded for analysis because they did not show a stable baseline fluorescence, because the onset of change did not coincide with the time of stimulation, or because the observed changes exceeded 2SD from the average response (n = 20, 9, 36, and 35 ROIs, respectively)*.

**Figure 3:**
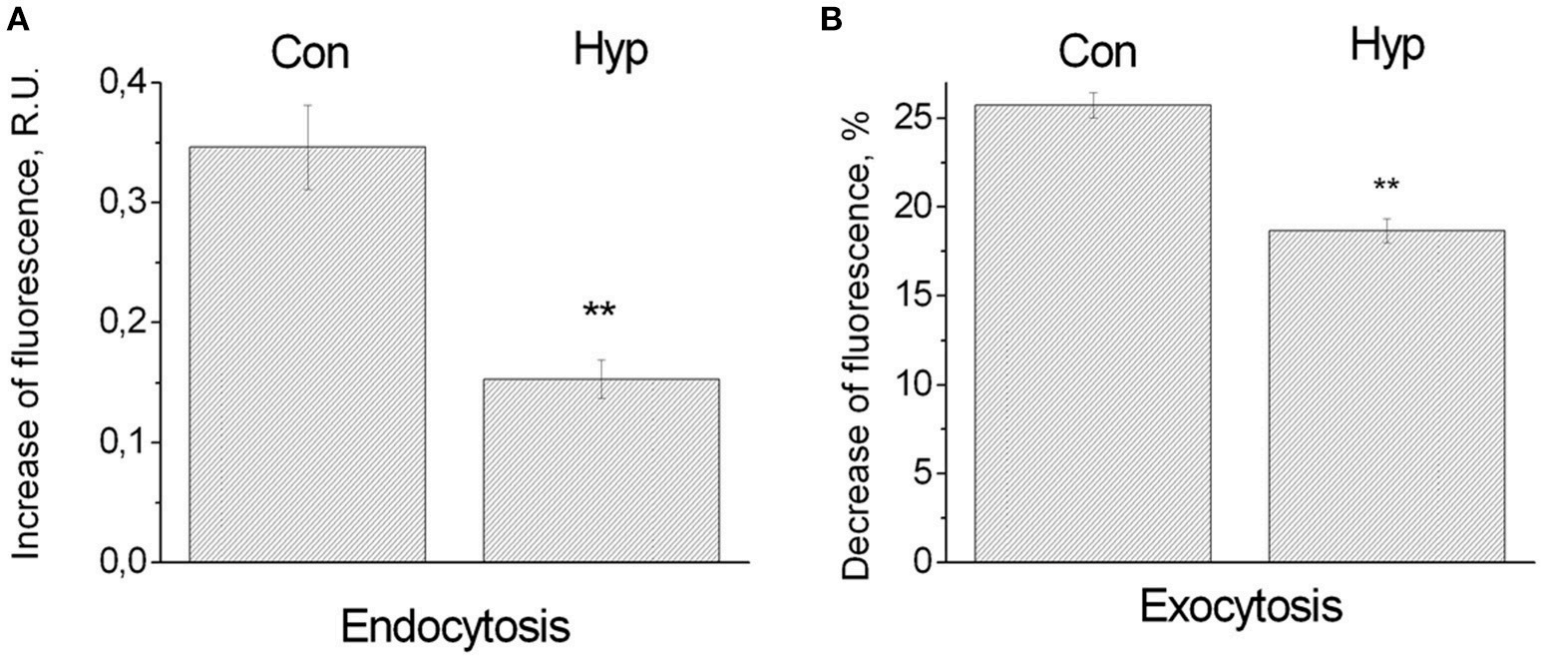
**Influence of hypoxia on the synaptic vesicle cycle. (A)** Endocytosis. Bars indicate increasing fluorescence upon a series of stimulation induced action potentials, in random units (R.U.), under normoxic conditions (258 ROIs in 11 cultures: Con), or hypoxic conditions (101 ROIs in 8 cultures: Hyp). Error bars indicate SEM, and reflect differences between ROIs. **(B)** Exocytosis. After loading, extracellular dye was washed out to measure the decreasing fluorescence upon a series of action potentials in response to stimulation. Decreasing fluoresce was normalized to the average fluorescence after dye loading. Error bars indicate SEM and reflect differences between 526 ROIs of 14 cultures (control conditions: Con), or 452 ROIs in 12 cultures (hypoxic conditions: Hyp). ^**^Indicate significant differences (*p* < 0.01).

Under control conditions endocytosis was 0.35 ± 0.035 R.U. (11 coverslips, 258 ROIs), under hypoxic conditions it dropped to 0.153 ± 0.016 R.U. (8 coverslips, 101 ROIs) (Figure 3A). Thus, endocytosis under hypoxic conditions was significantly less than under normoxic conditions (44%; *P* ≤ 0.01). Under control conditions exocytosis was 25.7 ± 0.7% or 2.92 ± 0.12 R.U. (14 coverslips, 526 ROIs), under hypoxic conditions it dropped to 18.66 ± 0.69% or 2.12 ± 0.12. R.U. (12 coverslips, 452 ROIs) (Figure 3). Thus, exocytosis also decreased significantly (to 72% of the normoxic value; *P* ≤ 0.01), but this decrease was less pronounced than the decrease seen with endocytosis.

## Discussion

In the rat brain, approximately half of the energy used for signaling is for synaptic neurotransmission, which makes synaptic function viable for effects of energy depletion (Attwell and Laughlin, 2001). Hypoxia has been shown to quickly depress synaptic functioning *in vivo* (Bolay et al., 2002; Sun et al., 2002), and in cortical *in vitro* preparations (Hofmeijer and van Putten, 2012; le Feber et al., 2016), far before the neurons start to deteriorate. Bolay et al showed that synaptic dysfunction was most likely caused by presynaptic mechanisms.

In most regions of interest (ROIs) we were able to obtain a stable baseline fluorescence, the vast majority of changes occurred at the onset of stimulation, and most changes were in the anticipated direction under both imaging conditions (endocytosis or exocytosis). Occasionally, we observed changes in the unexpected direction. Because we did not use a perfusion system it is possible that in case of exocytosis imaging, excess of released dye was taken up by other boutons through endocytosis. To avoid misinterpretation, the analyst, though blinded to the hypoxic condition, knew the imaging condition. In general, hypoxia impeded different stages of the synaptic vesicle cycle. Endocytosis seems to be more vulnerable to hypoxia, which is in good agreement with the notion that endocytosis is extremely sensitive to ATP depletion and metabolic disturbances (Rangaraju et al., 2014; Pathak et al., 2015; Hrynevich et al., 2016). Reduced endocytosis and exocytosis clearly occur after 6 h of hypoxia at pO_2_ ≈20 mmHg. At this hypoxic depth and duration, synaptic neurotransmission is disturbed, but individual neuronal functioning is not significantly affected, yet (le Feber et al., 2016). Since these processes are not independent, reduced exocytosis might be secondary to impeded endocytosis, or vice versa. The observation that endocytosis dropped much deeper than exocytosis suggests that reduced exocytosis may be secondary to reduced endocytosis, whereas a causal relation in the opposite direction seems less likely.

The ATP-sensitive steps that hamper endocytosis when ATP supply is compromised are not well-known (Rangaraju et al., 2014). Possible mechanisms include the ATP dependent disassembly and sorting of SNARE complex, which permits the fused membrane to enter the endocytic pathway (Heidelberger et al., 2002).

The effect of endocytosis inhibitors like dynasore on exocytosis and electrophysiological activity in comparison to the effect of hypoxia may further reveal whether reduced exocytosis occurs secondary to impeded endocytosis. Alternatively, energy depletion might directly affect exocytosis, e.g., through reduced phosphorylation of synapsin I, which impedes the transfer of synaptic vesicles from the reserve pool to the readily releasable pool and thus exocytosis. Dual staining for synapsin I and phosphosynapsin may reveal the relevance of this potential mechanism.

Ongoing spontaneous activity might severely hamper quantification of endocytosis and exocytosis. In the current study we used glutamatergic antagonists to block excitatory coupling between the neurons. Consequently, spontaneous activity dropped to (nearly) zero, and occurrence of exo/endocytosis was restricted to the episodes of stimulation. The late (synaptically driven) phase of the stimulus response (latencies > 15 ms), but not the early phase (0–15 ms) was blocked by application of APV (an NMDA receptor antagonist) and CNQX (an AMPA receptor antagonist) (Marom and Shahaf, 2002; Wagenaar et al., 2004). Stimulus responses confirmed the efficacy of the applied blockers (Figure 1C). Although it is common practice in imaging of synaptic vesicle recycling (Stevens and Williams, 2000; Burrone et al., 2006; Orenbuch et al., 2012), the efficacy of electrical stimulation largely dropped due to the application of these blockers. Whereas electrical stimulation under control conditions usually triggered a network burst, after glutamatergic blockade it only directly activated a small subset of neurons. This probably explains the relatively small response to electrical stimulation.

We therefore used potassium to induce depolarization of the plasma membrane and synaptic vesicle recycling. In neurons, the plasma membrane potential is determined by the potassium gradient across the membrane. Therefore, depolarization quickly follows an increase of the extracellular K^+^ concentration. This approach has the advantage that we can investigate the direct influence of hypoxia on synaptic vesicle recycling, avoiding the influence of possible potential changes across the plasma membrane or effects on voltage-gated sodium and potassium channels during shortage of oxygen (Müller and Somjen, 2000; Ayata and Lauritzen, 2015). Initial fluorescence increases in all endocytosis experiments were limited, and could certainly not account for the level of fluorescence at the beginning of subsequent exocytosis experiments, as illustrated in Figure 2. Additional dye was probably endocytosed at the time of washing between endocytosis and exocytosis measurements. During pipetting more dye reached the neurons, which were depolarized by the high potassium. This further increased the FM1-43 fluorescence to the level at which exocytosis measurement started. Still, we used the initial fluorescence increase to compare endocytosis under normoxic and hypoxic conditions. This was possible because this phenomenon occurred under both conditions and we had no reason to believe that it was differently affected by either condition.

Neurotransmitter release depends not only on synaptic vesicle recycling. It also can be dramatically changed by energy depletion induced collapse of resting membrane potential or modification of action potential. Our results clearly show that ATP-dependent steps of synaptic vesicle recycling are involved in synaptic failure during hypoxia. Potentially they can be targets for therapeutic intervention.

### ROI selection

Manual selection of ROIs is always a bit subjective. However, dye remnants after washing may inadvertently stain the parts of the plasma membrane, or even cell debris. Dye at these locations will respond only in a nonspecific way. Selection of ROIs diminishes the possible contribution of dye at these locations. In addition, selection of ROIs reduces the influence of areas that don't contain boutons. These areas generally reduce fluorescence, as well as fluorescence changes. Thus, they reduce the visualization of endo- and exocytosis, and possible alterations induced by hypoxia. Therefore, we tried to position the borders of ROIs closely around boutons (see Figure 2A). Imperfect positioning of the borders might affect the outcome only if areas without boutons were significantly different under hypoxic and control conditions. However, ROI positioning was done blinded to the condition, and given the large number of ROIs per condition it is very improbable that ROIs in one condition contained significantly more areas not containing boutons.

### Limitations of study

The use of dissociated neurons on multi electrode arrays supports the long term vitality of cultures, but has the disadvantage that cell cultures lack the typical structures found in the *in vivo* cortex. However, this study focused on generic aspects of synaptic functioning and synaptic vesicle recycling during hypoxia, which does not depend on a specific brain structure.

Another possible limitation lies in the interpretation of hypoxic depth and results from differences between the *in vivo* and *in vitro* ranges of normoxia and hypoxia. *In vivo* studies reported pO_2_ ≈30–35 mmHg during normoxia (Nair et al., 1987; Grote et al., 1996), much lower than normoxia in the current study. Dissociated cortical neurons are usually cultured under high partial oxygen pressures which are interpreted as “normoxic” conditions. It is unclear how this relatively high pO_2_ relates to the physiological oxygen pressure *in vivo*. However, minor pO_2_ drops immediately affected the activity of cultures, even while remaining far above the *in vivo* normoxia level of 30–35 mmHg (Hofmeijer et al., 2014; le Feber et al., 2016). Furthermore, we chose to restrict the available amount of oxygen but not glucose, whereas circulation problems generally confine the availability of both. *In vivo*, additional processes, possibly related to the limited availability of glucose, may occur in parallel to the processes observed in our model system.

In summary, our results demonstrate that hypoxia in cultured cortical networks rapidly depresses synaptic vesicle endocytosis and, to a lesser extent, exocytosis. Blocking of endocytosis has been shown to lead to short-term synaptic depression (Hua et al., 2013). These findings are in agreement with earlier research that showed that synaptic failure occurs quickly after the induction of hypoxia (Hofmeijer et al., 2014; le Feber et al., 2016), and that the failing processes are at least in part presynaptic (Bolay et al., 2002).

## Author contributions

SF: Substantial contributions to conception, cell culture experiments, analysis and interpretation of data, and writing of the manuscript. JH: Contribution to conception, and editing of the manuscript. Mv: Contribution to conception, and editing of the manuscript. JL: Substantial contributions to conception, analysis and interpretation of data, project management, and writing of the manuscript.

### Conflict of interest statement

The authors declare that the research was conducted in the absence of any commercial or financial relationships that could be construed as a potential conflict of interest.

## References

[B1] Attwell D. Laughlin S. B. (2001). An energy budget for signaling in the grey matter of the brain. J. Cereb. Blood Flow Metab. 21, 1133–1145. 10.1097/00004647-200110000-00001 11598490

[B2] Ayata C. Lauritzen M. (2015). Spreading depression, spreading depolarizations, and the cerebral vasculature. Physiol. Rev. 95, 953–993. 10.1152/physrev.00027.2014 26133935 PMC4491545

[B3] Bolay H. Gürsoy-Özdemir Y. Sara Y. Onur R. Can A. Dalkara T. (2002). Persistent defect in transmitter release and synapsin phosphorylation in cerebral cortex after transient moderate ischemic injury. Stroke 33, 1369–1375. 10.1161/01.str.0000013708.54623.de 11988617

[B4] Burrone J. Li Z. Murthy V. N. (2006). Studying vesicle cycling in presynaptic terminals using the genetically encoded probe synaptopHluorin. Nat. Prot. 1, 2970–2978. 10.1038/nprot.2006.449 17406557

[B5] Cochilla A. J. Angleson J. K. Betz W. J. (1999). Monitoring secretory membrane with FM1-43 fluorescence. Annu. Rev. Neurosci. 22, 1–10. 10.1146/annurev.neuro.22.1.1 10202529

[B6] Dittman J. Ryan T. A. (2009). Molecular circuitry of endocytosis at nerve terminals. Annu. Rev. Cell Dev. Biol. 25, 133–160. 10.1146/annurev.cellbio.042308.113302 19575674

[B7] Gaffield M. A. Betz W. J. (2006). Imaging synaptic vesicles exocytosis and endocytosis with FM dyes. Nat. Protoc. 1, 2916–2921. 10.1038/nprot.2006.476 17406552

[B8] Gao T. M. Pulsinelli W. A. Xu Z. C. (1999). Changes in membrane properties of CA1 pyramidal neurons after transient forebrain ischemia *in vivo*. Neuroscience 90, 771–780. 10.1016/S0306-4522(98)00493-X 10218778

[B9] George P. M. Steinberg G. K. (2015). Novel stroke therapeutics: unraveling stroke pathophysiology and its impact on clinical treatments. Neuron 87, 297–309. 10.1016/j.neuron.2015.05.041 26182415 PMC4911814

[B10] Grote J. Laue O. Eiring P. Wehler M. (1996). Evaluation of brain tissue O_2_ supply based on results of PO_2_ measurements with needle and surface microelectrodes. J. Auton. Nerv. Syst. 57, 168–172. 10.1016/0165-1838(95)00096-8 8964943

[B11] Heidelberger R. Sterling P. Mathews G. (2002). Roles of ATP in depletion and replenishment of the releasable pool of synaptic vesicles. J. Neurophysiol. 88, 98–106. 12091535 10.1152/jn.2002.88.1.98

[B12] Hochachka P. W. Buck L. T. Doll C. J. Land S. C. (1996). Unifying theory of hypoxia tolerance: molecular/metabolic defense and rescue mechanisms for surviving oxygen lack. Proc. Natl. Acad. Sci. U.S.A. 93, 9493–9498. 10.1073/pnas.93.18.9493 8790358 PMC38456

[B13] Hofmeijer J. Mulder A. T. Farinha A. C. vanPutten M. J. A. M. le Feber J. (2014). Mild hypoxia affects synaptic connectivity incultured neuronal networks. Brain Res. 1557, 180–189. 10.1016/j.brainres.2014.02.027 24560899

[B14] Hofmeijer J. van Putten M. J. A. M. (2012). Ischemic cerebral damage. Stroke 43, 607–615. 10.1161/STROKEAHA.111.632943 22207505

[B15] Hrynevich S. V. Waseem T. V. Hebert A. Pellerin L. Fedorovich S. V. (2016). beta-Hydroxybutyrate supports synaptic vesicle cycling but reduces endocytosis and exocytosis in rat brain synaptosomes. Neurochem. Int. 93, 73–81. 10.1016/j.neuint.2015.12.014 26748385

[B16] Hua Y. Woehler A. Kahms M. Haucke V. Neher E. Klingauf J. (2013). Blocking endocytosis enhances short-term synaptic depression under conditions of normal availability of vesicles. Neuron 80, 343–349. 10.1016/j.neuron.2013.08.010 24139039

[B17] Klingauf J. Kavalali E. T. Tsien R. W. (1998). Kinetics and regulation of fast endocytosis at hippocampal synapses. Nature 394, 581–585. 10.1038/29079 9707119

[B18] le Feber J. Tzafi Pavlidou S. Erkamp N. van Putten M. J. A. M. Hofmeijer J. (2016). Progression of neuronal damage in an *In Vitro* model of the Ischemic, Penumbra. PLoS ONE 11:e0147231. 10.1371/journal.pone.0147231 26871437 PMC4752264

[B19] Marom S. Shahaf G. (2002). Development, learning and memory in large random networks of cortical neurons: lessons beyond anatomy. Q. Rev. Biophys. 35, 63–87. 10.1017/S0033583501003742 11997981

[B20] Müller M. Somjen G. G. (2000). Na^+^ dependence and the role of glutamate receptors and Na^+^ channels in ion fluxes during hypoxia of rat hippocampal slices. J. Neurophysiol. 84, 1869–1880. 11024079 10.1152/jn.2000.84.4.1869

[B21] Nair P. K. Buerk D. G. Halsey J. H. Jr. (1987). Comparisons of oxygen metabolism and Tissue pO2 in cortex and hippocampus of Gerbil Brain. Stroke 18, 616–622. 10.1161/01.STR.18.3.616 3590255

[B22] Orenbuch A. Shalev L. Marra V. Sinai I. Lavy Y. Kahn J.. (2012). Synapsin selectively controls the mobility of resting pool vesicles at hippocampal terminals. J. Neurosci. 32, 3969–3980. 10.1523/jneurosci.5058-11.2012 22442064 PMC3492757

[B23] Pathak D. Shields L. Y. Mendelsohn B. A. Haddad D. Lin W. Gerencser A. A.. (2015). The role of mitochondrially derived ATP in synaptic vesicle recycling. J. Biol. Chem. 290, 22325–22336. 10.1074/jbc.m115.656405 26126824 PMC4566209

[B24] Rangaraju V. Calloway N. Ryan T. A. (2014). Activity-driven local ATP synthesis is required for synaptic function. Cell 156, 825–835. 10.1016/j.cell.2013.12.042 24529383 PMC3955179

[B25] Romijn H. J. van Huizen F. Wolters P. S. (1984). Towards an improved serum-free, chemically defined medium for long-term culturing of cerebral cortex tissue. Neurosci. Biobehav. Rev. 8, 301–334. 10.1016/0149-7634(84)90055-1 6504415

[B26] Stevens C. F. Williams J. H. (2000). “Kiss and run” exocytosis at hippocampal synapses. Proc. Natl. Acad. Sci. U.S.A. 97, 12828–12833. 10.1073/pnas.230438697 11050187 PMC18849

[B27] Südhof T. C. (2004). The synaptic vesicle cycle. Annu. Rev. Neurosci. 27, 509–547. 10.1146/annurev.neuro.26.041002.131412 15217342

[B28] Südhof T. C. (2013). Neurotransmitter release: the last millisecond in the life of a synaptic vesicle. Neuron 80, 675–690. 10.1016/j.neuron.2013.10.022 24183019 PMC3866025

[B29] Sun M.-K. Xu H. Alkon D. L. (2002). Pharmacological protection of synaptic function, spatial learning, and memory from transient hypoxia in rats. J. Pharmacol. Exp. Ther. 300, 408–416. 10.1124/jpet.300.2.408 11805198

[B30] Wagenaar D. A. Pine J. Potter S. M. (2004). Effective parameters for stimulation of dissociated cultures using multi-electrode arrays. J. Neurosci. Methods 138, 27–37. 10.1016/j.jneumeth.2004.03.005 15325108

